# Real-world evidences on adjuvant Pembrolizumab for renal cell carcinoma: results from the multicenter real-world ARON-1 study

**DOI:** 10.1007/s00262-025-04230-w

**Published:** 2025-11-14

**Authors:** Ray Manneh Kopp, Francesco Massari, Enrique Grande, Timothy J. Schieber, Yüksel Ürün, Jindřich Kopecký, Javier Molina-Cerrillo, Umberto Basso, Jakub Kucharz, Thomas Büttner, Renate Pichler, Ondrej Fiala, Zin W. Myint, Luca Galli, Tomas Buchler, Mimma Rizzo, Hatice Bolek, Maria T. Bourlon, Vincenza Conteduca, Alvaro Pinto, Alessandro Rizzo, Matteo Rosellini, Giandomenico Roviello, Anca Zgura, Veronica Mollica, Antonia Partl, Ahmet Yildirim, Umut Akova, Sebastiano Buti, Fernando Sabino Marques Monteiro, Andrey Soares, Camillo Porta, Mehmet Asim Bilen, Haoran Li, Matteo Santoni

**Affiliations:** 1 Clinical Oncology, Sociedad de Oncología y Hematología del Cesar, Valledupar, Colombia; 2 Medical Oncology, IRCCS Azienda Ospedaliero-Universitaria Di Bologna, Bologna, Italy; 3 Department of Medical and Surgical Sciences (DIMEC), University of Bologna, Bologna, Italy; 4 Department of Medical Oncology, MD Anderson Cancer Center Madrid, Madrid, Spain; 5 Division of Medical Oncology, Department of Internal Medicine, University of Kansas Cancer Center, Kansas city, USA; 6 Department of Medical Oncology, Faculty of Medicine, Ankara University, Türkiye; Westwood, Ankara, KS 06620 USA; 7 Department of Clinical Oncology and Radiotherapy, University Hospital Hradec Kralove, Hradec Kralove, Czechia; 8 Department of Medical Oncology, Hospital Ramón y Cajal, Madrid, Spain; 9 Oncology 1 Unit, Department of Oncology, Istituto Oncologico Veneto IOV IRCCS, Padua, Italy; 10 Department of Uro-Oncology, Maria Sklodowska-Curie National Research Institute of Oncology Warsaw, Warsaw, Poland; 11 Department of Urology, University Hospital Bonn (UKB), Bonn, Germany; 12 Department of Urology, Medical University of Innsbruck, Innsbruck, Austria; 13 Department of Oncology and Radiotherapeutics, Faculty of Medicine, University Hospital in Pilsen, Charles University, Pilsen, Czech Republic; 14 Biomedical Center, Faculty of Medicine in Pilsen, Charles University, Pilsen, Czech Republic; 15 Division of Medical Oncology, Department of Internal Medicine, Markey Cancer Center, University of Kentucky, Lexington, KY USA; 16 Oncology Unit 2, University Hospital of Pisa, 56126 Pisa, Italy; 17 Department of Oncology, Second Faculty of Medicine, Charles University and University Hospital Motol, V Uvalu 84, 150 06, Prague, Czech Republic; 18 Medical Oncology Unit, Azienda Ospedaliera Universitaria Consorziale Policlinico Di Bari, Bari, Italy; 19 Instituto Nacional de Ciencias Médicas y Nutrición Salvador Zubirán, Mexico City, Mexico; 20 Unit of Medical Oncology and Biomolecular Therapy and C.R.E.A, T.E - Center for Research and Innovation Medicine, Department. of Medical and Surgical Sciences, University of Foggia, Policlinico Riuniti, Foggia, Italy; 21 Servicio de Oncología, Hospital Universitario La Paz, Madrid, Spain; 22 S.S.D. C.O.R.O. Bed Management Presa in Carico, TDM, IRCCS Istituto Tumori ‘‘Giovanni Paolo II’’, Viale Orazio Flacco 65, 70124 Bari, Italy; 23 Department of Health Sciences, Section of Clinical Pharmacology and Oncology, University of Florence, Florence, Italy; 24 Department of Oncology-Radiotherapy, Alexandru Trestioreanu Institute of Oncology, ‘‘Carol Davila’’ University of Medicine and Pharmacy, 020021 Bucharest, Romania; 25 Department of Hematology and Oncology, Emory University School of Medicine, Atlanta, GA USA; 26 Medical Oncology Unit, University Hospital of Parma, Parma, Italy; 27 Department of Medicine and Surgery, University of Parma, Parma, Italy; 28 Oncology and Hematology Department, Hospital Sírio Libanês, Brasília, Brazil; 29 Latin American Cooperative Oncology Group - LACOG, Porto Alegre, Brazil; 30 Oncology Unit, Hospital Israelita Albert Einstein, São Paulo, SP Brazil; 31 Interdisciplinary Department of Medicine, University of Bari ‘‘Aldo Moro’’, Bari, Italy; 32 Winship Cancer Institute of Emory University, Atlanta, GA USA; 33 Medical Oncology Unit, Macerata Hospital, Macerata, Italy

**Keywords:** Adjuvant therapy, ARON-1, Immune checkpoint inhibitor, Pembrolizumab, Real-world data, Renal cell carcinoma

## Abstract

**Background:**

Pembrolizumab has demonstrated efficacy in improving disease-free survival (DFS) and overall survival (OS) as adjuvant therapy in clear cell renal cell carcinoma (ccRCC) at a higher risk of recurrence. However, real-world data on its effectiveness and safety remain limited. This study evaluates DFS, OS and severe adverse events (SAEs) associated with adjuvant pembrolizumab in a multicenter international cohort.

**Methods:**

This retrospective analysis included 311 ccRCC patients treated with adjuvant pembrolizumab across 40 hospitals in 12 countries from the ARON-1 dataset. Eligible patients had histologically confirmed ccRCC with high relapse risk and received up to 17 cycles of pembrolizumab. The primary objective was DFS, with OS and safety as secondary objectives. Kaplan–Meier survival estimates, Cox proportional hazards models and log-rank tests were used for statistical analysis.

**Results:**

At a median follow-up of 15.4-month, 2-year OS and DFS rates were 95% and 69%, respectively. Recurrence occurred in 20% of patients, primarily in the lungs (11%) and bones (5%). DFS was significantly impaired in patients < 65 years (HR 2.14, *p* = 0.005), N1 disease (HR 5.42, *p* = 0.004) and sarcomatoid dedifferentiation (HR 2.54, *p* = 0.007). SAEs led to 19% treatment discontinuation, with colitis (4%), hypertransaminasemia (4%) and nephritis (3%) as the most common events. The study’s retrospective nature and short follow-up limit long-term outcome assessments.

**Conclusions:**

This large real-world study confirms pembrolizumab’s effectiveness and manageable safety profile in the adjuvant setting for intermediate-high and high-risk ccRCC. Further research is needed to refine patient selection strategies and evaluate long-term outcomes.

**Supplementary Information:**

The online version contains supplementary material available at 10.1007/s00262-025-04230-w.

## Introduction

In 2020, about 430,000 new cases of kidney cancer and 180,000 related deaths have been reported [1]. The majority of kidney malignancies diagnosed in the overall population is represented by renal cell carcinoma (RCC), which constitutes a heterogeneous group of cancers: While clear cell RCC (ccRCC) accounts for approximately 80% of cases, the remaining 20% may be categorized as non-clear cell RCC (nccRCC), by including a variety of histological subtypes with specific molecular features and cytogenetic alterations [2].

The standard of care for localized RCC is represented by a surgical approach, based on radical or partial nephrectomy, regardless of the histological subtype (ccRCC *vs* nccRCC) [3]. However, despite surgical resection, localized ccRCC characterized by some risk factors carries a 5-year recurrence rate of up to 68% [3]. As a matter of fact, a higher risk of recurrence appears to be related with features as tumor stage 2 with nuclear grade 4, tumor stage ≥ 3, the presence of sarcomatoid dedifferentiation, the presence of regional lymph node metastases or oligometastatic disease at the time of radical nephrectomy, with or without metastasectomy [4–6]. Several scores have been developed and validated in order to help clinicians to identify tumors at a high risk of recurrence to be treated with adjuvant intent.

A meaningful revolution in the management of ccRCC has occurred in the last decade with the approval and use of cancer immunotherapy, based on the administration of immune checkpoint inhibitors (ICIs). The therapeutic benefits of immunotherapy were recently shown not to be strictly confined to patients with a metastatic staged disease but also in selected patients with localized and locally advanced ccRCC [6, 7].

During the previous era of anti-vascular endothelial growth factor (VEGF) tyrosine-kinase inhibitors (TKIs), adjuvant strategies using these drugs did not demonstrate survival benefits for patients with a higher risk of relapse in clinical trials, thus not allowing their effective use in everyday clinical practice [8]. The breakthrough in the perioperative management of localized ccRCC came with the publication of the phase III KEYNOTE-564 study’s results [6]. The use of the anti-programmed cell death-1 (PD-1) pembrolizumab as an adjuvant treatment strategy after radical surgery showed both statistically significant, as well clinically meaningful, improved disease-free survival (DFS) along with a meaningful but modest benefit in terms of overall survival (OS) when compared with placebo in patients with ccRCC at a higher risk of relapse [9]. On the other hand, other randomized trials investigating a perioperative or adjuvant treatment based on ICIs have not demonstrated so far significant improvements in survival outcomes. This latter variability in outcomes among these studies may be related to differences in therapy duration, patient eligibility criteria or the different type of ICI administered (anti-PD-1 or -PD-L1) [10].

Additionally, the lack of reliable predictive biomarkers significantly hinders the ability to select patients who are most likely to benefit from this therapeutic approach [11, 12]. Furthermore, while the initial pre-planned analysis showed a more pronounced DFS benefit in M1 NED patients [6], the latest survival outcomes analysis highlights the greatest OS benefit in intermediate-to-high-risk ccRCC patients [9, 11], who represent a potentially broad and heterogeneous population. Consequently, identifying as many patients as possible who are likely to derive the greatest benefit from adjuvant pembrolizumab is nowadays crucial, and real-world data may play a critical role in addressing these needs and providing valuable insights.

ARON-1 (NCT05287464) is a global project analyzing real-world data from patients with RCC across multiple centers worldwide [13–17]. As part of the ARON-1 initiative, we conducted a multicenter retrospective analysis of treatment outcomes in patients with resected ccRCC at a higher risk of relapse who received adjuvant pembrolizumab. This analysis included data from 40 centers across 12 countries.

## Patients and methods

### Study population

This retrospective analysis included patients aged 18 years and older, all of whom had a histologically confirmed diagnosis of ccRCC as confirmed through histological reports, who received adjuvant pembrolizumab for an increased risk of recurrence. Data were gathered from individuals treated between December 1, 2021, and December 1, 2024, across 40 medical centers in 12 countries (see Figure S1).

Pembrolizumab adjuvant therapy continued until disease recurrence and up to 17 cycles, development of intolerable side effects or patient death. Imaging studies, including contrast-enhanced computed tomography (CT) or magnetic resonance imaging (MRI), were performed every 8–12 weeks according to local protocols. Routine physical exams and laboratory tests were conducted every 3–6 weeks, adhering to local guidelines.

Data for this study were collected retrospectively from patient records (either electronic or paper), including variables such as age, sex, type of nephrectomy, type and time of recurrence, treatment-related adverse events, efficacy outcomes and subsequent treatments following adjuvant therapy. Patients without adequate data for tumor assessment or those lost to follow-up were excluded from the analysis. Disease risk category followed the criteria from the KEYNOTE-564 study [6].

### Study endpoints

The primary endpoint of this ARON-1 substudy was to assess the real-world DFS, defined as the time from treatment initiation to the first documented local or distant recurrence, of adjuvant pembrolizumab in patients with ccRCC. The secondary endpoint was OS, defined as the time from the start of pembrolizumab until death from any cause. Additional secondary endpoint included safety and tolerability, DFS by clinical-pathological features, local DFS (L-DFS), defined as the time from treatment initiation to the first documented local recurrence and distant DFS (D-DFS), as the time from treatment initiation to the first documented distant recurrence.

Patients who did not recur or were lost to follow-up at the time of analysis were censored based on their most recent follow-up data.

Severe adverse events (SAEs) were defined according to the Common Terminology Criteria for Adverse Events (CTCAE) v5.0.

### Statistical analysis

Statistical analyses were performed using MedCalc software (version 19.6.4, MedCalc Software, Broekstraat 52, 9030 Mariakerke, Belgium). Kaplan–Meier methods were used to estimate OS and DFS, and 95% confidence intervals (95% CI) were calculated using Rothman’s method. The log-rank test was applied to compare survival distributions.

Confidence intervals (95% CI) were calculated by Rothman’s formula. Cox proportional hazards models, using Schoenfeld residuals, were applied basing on univariable significance to assess the multivariable impact on patient survival, providing hazard ratios (HRs) and 95% confidence intervals (CIs), including clinical and pathological variables related to patients’ outcomes: sex, age ≥ 65y (in accordance with KEYNOTE-564 cutoff), type of surgical approach (partial vs. radical nephrectomy), sarcomatoid dedifferentiation and disease risk category according to the criteria from the KEYNOTE-564 study [6]. The Fisher’s exact test was used for pairwise comparisons of categorical variables, and chi-square tests were applied for multiple categorical comparisons. A *p*-value of < 0.05 was considered statistically significant, and all p-values were two-sided.

### Ethics approval

Ethical approval for the ARON-1 study was obtained from the Ethics Committee of the Marche Region (2021–492) and the Institutional Review Boards of all participating centers, in accordance with relevant regulations in each country. The study adhered to Good Clinical Practice (GCP) guidelines and international ethical standards for biomedical research. All protocols followed the ethical principles outlined in the Declaration of Helsinki for human research.

## Results

Three hundred and eleven patients treated with adjuvant pembrolizumab were included from the ARON-1 dataset (Figure S2). The median age was 61 years (range 25–85 years), with 74% of male patients and 51% of patients treated in Europe.

Nephrectomy was partial in 5% and radical in 95% of cases: 257 patients (83%) presented a T3 stage at diagnosis. Tumor nuclear grading was G4 in 84 patients (27%), with 17% of cases reporting sarcomatoid dedifferentiation. Five percent of patients exhibited lymph node involvement (N1) at the time of diagnosis, while 11% were disease-free following nephrectomy and metastasectomy (M1 NED). The complete list of patients’ characteristics is reported in Table 1.

**Table 1:** Patients’ characteristics

Characteristics	Overall no. (%)
Total patients	311 (100)
Median age (range) < 65y	61 (25–85) 196 (63)
**Gender** Male Female	229 (74) 82 (26)
Geographical location North America Europe Rest of the world	102 (33) 159 (51) 50 (16)
Type of nephrectomy Partial Radical	14 (5) 297 (95)
Primary tumor pathological stage T1 T2 T3 T4	14 (5) 25 (8) 257 (83) 15 (4)
Tumor nuclear grading 1 2 3 4	4 (1) 80 (26) 143 (46) 84 (27)
Lymph node pathological stage N0 N1	294 (95) 17 (5)
Metastatic stage M0 M1 with no evidence of disease	277 (89) 34 (11)
Disease risk category M0 (intermediate to high) M0 (high) M1 with no evidence of disease	250 (80) 27 (9) 34 (11)
Sarcomatoid dedifferentiation	54 (17)

### Survival Analysis

**Fig. 1:**
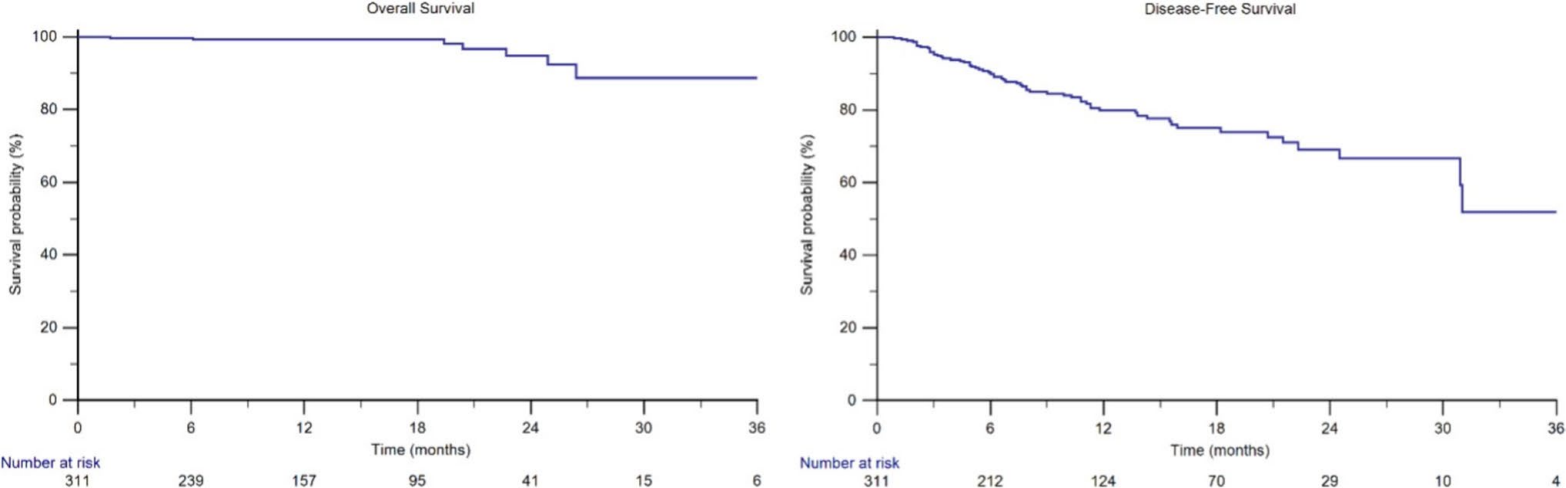
Overall survival and disease-free survival in RCC patients receiving adjuvant pembrolizumab

The median follow-up was 15.4 months (95%CI 11.2–18.8). The median OS was not reached (NR), with 99% of 1-year OS rate and 95% of 2-year OS rate (Fig. 1).

The median DFS was NR, with 90%, 80%, 75% and 69% of 6-month, 1-year, 18-month and 2-year DFS rates, respectively (Fig. 1). Sixty-one patients (20%) recurred during or after adjuvant pembrolizumab therapy, with 5 exclusive local recurrences (2%), 48 exclusive distant recurrences (15%) and 8 concomitant local and distant recurrences (3%). Sites of metastases in recurrent patients are shown in Table S1, Supplementary Materials.

**Fig. 2:**
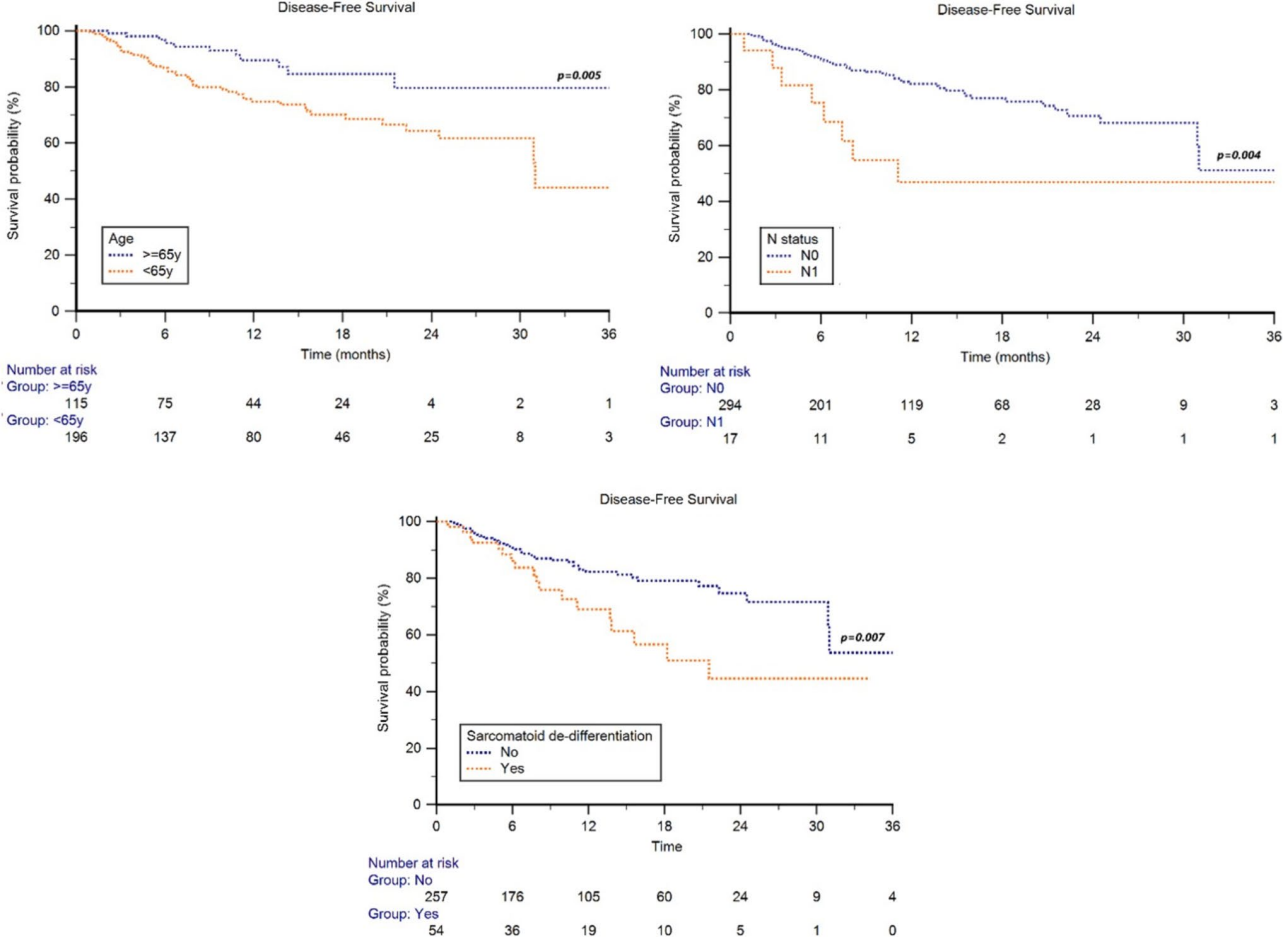
Disease-free survival in RCC patients receiving adjuvant pembrolizumab stratified by clinicopathological features

The median DFS was reduced in patients aged < 65 years (NR *vs* NR, HR 2.14, 95%CI 1.26–3.63, *p* = 0.005, Fig. 2), while no significant differences were found between males and females (NR *vs* NR, HR 0.92, 95%CI 0.51–1.65, *p* = 0.772).

The median DFS was NR in patients with N0 lymph node stage and 11.2 months (95%CI 5.4–12.8) in the N1 subgroup (HR 5.42, 95%CI 1.72–17.1, *p* = 0.004, Fig. 2). The median DFS was also significantly impaired in patients with sarcomatoid dedifferentiation (21.5 months, 95%CI 17.1–25.8 *vs* NR, HR 2.54 1.29–4.98, *p* = 0.007, Fig. 2).

By stratifying patients by disease risk category, the median DFS was NR in M0 intermediate to high, M0 high and M1 NED patients (*p* = 0.316), with a 1-year DFS rate of 83%, 73% and 70% and a 2-year DFS rate of 71%, 60% and 56% in the three subgroups.

No statistically significant differences in terms of median DFS were found between patients who underwent radical or partial nephrectomy (NR *vs* NR, HR 2.08, 95%CI 0.55–7.48, *p* = 0.284).

L-DFS was 5.4 months (95%CI 3.0–31.0), with 65% recurred within 6 months, 20% between 6 months and 1 year and 15% beyond 1 year.

D-DFS was 6.7 months (95%CI 5.2–31.0), with 41% recurred within 6 months, 36% between 6 months and 1 year and 23% over 1 year.

### Prognostic factors

Sarcomatoid dedifferentiation was the only factor significantly associated with OS at univariable analysis (Table 2). Moreover, age < 65y and sarcomatoid dedifferentiation were significantly correlated with DFS at both univariable and multivariable analyses (Table 2).

**Table 2:** Prognostic factors in RCC patients treated with adjuvant pembrolizumab

Overall Survival	Univariable Cox Regression		Multivariable Cox Regression	
	HR (95%CI)	*p-value*	HR (95%CI)	*p-value*
Sex (females vs males)	0.48 (0.05 − 4.12)	0.499	–	–
Age (< 65y vs ≥ 65y)	0.84 (0.16 − 4.49)	0.842	–	**–**
Nephrectomy (partial vs radical)	1.02 (0.59 − 1.75)	0.950	–	–
Sarcomatoid differentiation (yes vs no)	5.05 (1.13 − 22.68)	**0.035**	–	**–**
Disease Risk Category (M1 NED vs M0 intermediate to high or high)	1.46 (0.17 − 12.23)	0.727	–	–
Disease-Free Survival	*Univariable Cox Regression*		*Multivariable Cox*	
	HR (95%CI)	*p-value*	HR (95%CI)	*p-value*
Sex (females vs males)	0.92 (0.50 − 1.67)	0.773	–	–
Age (< 65y vs ≥ 65y)	2.49 (1.29 − 4.78)	**0.006**	2.51 (1.30 − 4.83)	**0.006**
Nephrectomy (partial vs radical)	1.73 (0.63 − 4.80)	0.290		
Sarcomatoid differentiation (yes vs no)	2.10 (1.21 − 3.65)	**0.008**	2.13 (1.23 − 3.69)	**0.007**
Disease Risk Category (M1 NED vs M0 intermediate to high or high)	1.65 (0.84 − 3.26)	0.146	–	**–**

### Safety

Fifty-nine patients (19%) reported severe adverse events (SAEs), registering 1 SAE in 47 patients (14%) and > 1 SAEs in 12 patients (4%). Sixty-one patients discontinued pembrolizumab therapy, whose 59 (19%) discontinued due to SAEs, 1 (< 1%) for pregnancy and 1 for personal reasons (< 1%). The most common SAEs were colitis (4%), hypertransaminasemia (4%) and nephritis (3%). The complete list of SAEs is reported in Table 3.

**Table 3:** List of severe adverse events (SAEs) in RCC patients treated with adjuvant pembrolizumab

Characteristics	Overall no. (%)
Total number of patients	311 (100)
Total number of SAEs	71 (23)
Patients reporting SAEs Patients with 1 SAE Patients with > 1 SAEs	59 (19) 47 (14) 12 (4)
Therapy discontinuations	61 (20)
Therapy discontinuations due to SAEs	59 (19)
Colitis	12 (4)
Hepatopathy	11 (4)
Nephritis	8 (3)
Pneumonitis	7 (2)
Fatigue	5 (2)
Rash	4 (1)
Arthritis	4 (1)
Thyroiditis	3 (1)
Adrenal insufficiency	3 (1)
Cardiovascular toxicity	3 (1)
Pancreatitis	3 (1)
Sjogren syndrome	2 (1)
Myositis	2 (1)
Anemia	1 (< 1)
Myasthenia	1 (< 1)
Nausea	1 (< 1)
Hypophysitis	1 (< 1)

**Fig. 3:**
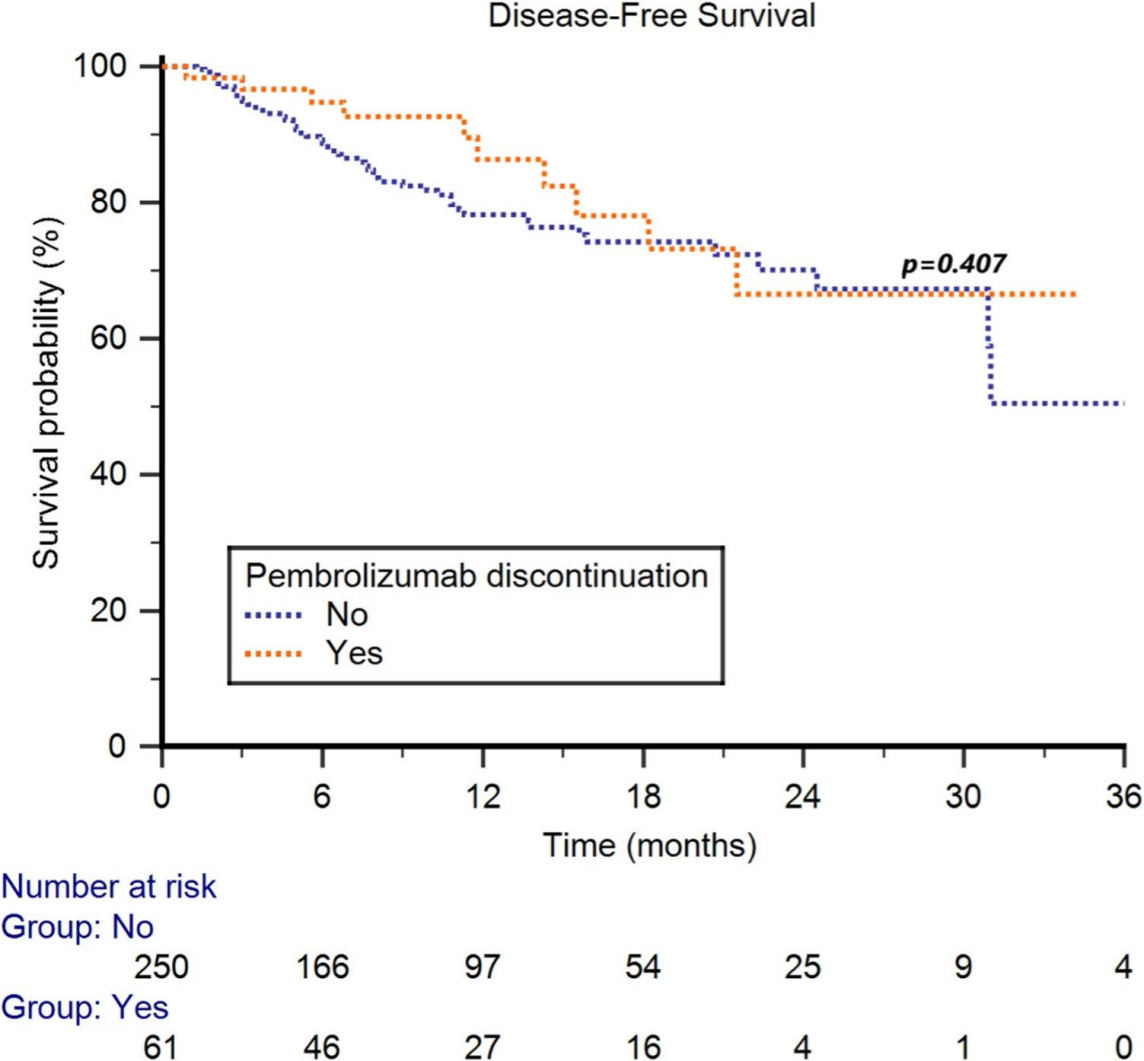
Disease-free survival in RCC who discontinued or not adjuvant pembrolizumab

No significant differences in terms of median DFS were reported between patients who discontinued pembrolizumab compared to subjects who did not discontinue (NR vs NR, HR 0.77, 95%CI 0.41–1.43, *p* = 0.407, Fig. 3).

## Discussion

With the growing number of clinical studies investigating novel therapeutic strategies for RCC, there has been a parallel rise in clinical reports discussing many adjuvant options in the localized/locally advanced disease [7].

In the previous TKI era, no study demonstrated a definitive role for targeted therapy as adjuvant strategy in resected ccRCC patients, except for the S-TRAC trial, which showed a modest DFS benefit with sunitinib but related to a high toxicity rate and no statistically significant benefit in OS [8]. More recently, the breakthrough came with the publication of the pivotal KEYNOTE-564 study’s results [6, 9, 11]. As a matter of fact, pembrolizumab is the only ICI to demonstrate a statistically significant benefit in both DFS and OS as adjuvant therapy in ccRCC [6, 9], whereas other immunotherapeutic agents have not achieved the same outcomes [7]. Nonetheless, real-world data are needed to further assess the role of pembrolizumab in this setting, especially considering that other immunotherapy approaches have failed in the same setting.

To our knowledge, the current ARON-1 analysis represents one of the largest cohorts to date evaluating the efficacy and safety of adjuvant pembrolizumab in ccRCC [18, 19], in a multicentric and international population. The substantial sample size allows for a comprehensive assessment of treatment outcomes on a large scale, providing valuable insights into its real-world effectiveness.

The population included in our dataset is mostly similar to that enrolled in the KEYNOTE-564, even though in our study 14 patients (5%) had T1 disease, which was not included in the phase III trial, and there was a higher percentage of M1 NED and sarcomatoid dedifferentiation (11% and 17% in our case series and 5.8% and 10.5% in the KEYNOTE-564 study, respectively).

Our findings are in line with the survival benefit observed in the pivotal KEYNOTE-564 trial [6, 9], with considerable 1-year and 2-year OS rates (99% and 95%, respectively) and promising DFS rates (90% at 6 months, 80% at 1 year and 69% at 2 years). Despite a lower median follow-up compared to the pivotal trial (15.4 months), these results appear to confirm the efficacy of pembrolizumab in preventing disease recurrence across different risk categories. In our dataset, 2-year DFS rate was 69%, slightly lower than reported in the KEYNOTE-564 (77.3%), presumably related to the real-world setting of our analysis and the higher percentage of M1 NED and sarcomatoid dedifferentiation, which are associated with a worse prognosis.

Our study showed a disease recurrence occurring in 20% of patients of our cohort, predominantly at distant sites. The impaired DFS observed in patients with lymph node involvement (N1) and sarcomatoid features aligns with prior studies suggesting these features as poor prognostic factors [4, 5, 11, 12]. In more details, patients with sarcomatoid dedifferentiation were characterized by a markedly reduced median DFS (21.5 months *vs.* NR, HR 2.54, *p* = 0.007), while those with N1 disease had a median DFS of 11.2 months. On the other hand, no significant DFS differences were found between patients undergoing radical versus partial nephrectomy, suggesting that the extent of surgery does not critically impact adjuvant therapy outcomes in this setting.

Interestingly, younger patients (< 65 years) had a significantly worse DFS compared to older patients (*p* = 0.005). Whereas this finding needs further investigation, it may reflect underlying biological differences in tumor behavior or treatment response between age groups. Notably, in the KEYNOTE-564 trial the benefit of pembrolizumab in the subgroup analysis of patients aged < 65 years was higher (HR for OS 0.51, 95% CI 0.31–0.83) [9]. Age and disease-free survival (DFS) in renal cell carcinoma (RCC) is a complex relationship with inconsistent findings across studies; some suggest age, particularly for younger patients or a specific cutoff like 60 years, can be a risk factor for shorter DFS, while others find no significant association, especially in certain RCC subtypes like translocation RCC [20]

As shown in Table S1 (Supplementary Materials), 11% of patients in our cohort experienced a pulmonary relapse, 5% experienced a skeletal relapse and about 5% developed endocrine glands metastases, while much rarer appeared to be hepatic and brain metastases in whom relapsed after adjuvant pembrolizumab. In some studies, RCC patients who experienced recurrence while on adjuvant immunotherapy or within three months of completing ICI-based treatment were shown to have a significantly higher incidence of bone metastases [21]. Bone metastases in RCC are typically associated with poorer survival outcomes [22] and are enriched in the angiogenic/stromal molecular subgroups, which may contribute to an ICI-resistant tumor microenvironment [22–24]. Furthermore, RCC exhibits site-specific metastatic tropism, and it remains unknown whether adjuvant ICI-based therapy influences progression patterns and metastatic site preference [25]. Additionally, approximately 5% of relapsed patients in our cohort developed disease in sites traditionally associated with a favorable prognosis, such as the adrenal glands, thyroid and pancreas [26, 27] (Table S1, Supplementary Materials). Many studies have shown that these endocrine glands metastases were characterized by a higher expression of genes involved in the angiogenesis pathway, thus correlating with a potential increased sensitivity to VEGF TKIs [23, 24].

Regarding the safety profile, pembrolizumab was generally well tolerated in out cohort, with SAEs occurring in 19% of all the patients, being primarily represented by immune-related colitis (4%), hepatitis (4%) and nephritis (3%). These safety outcomes are consistent with previous clinical trial data and highlight the manageable toxicity profile of pembrolizumab in a real-world setting [9]. Of note, treatment discontinuation due to SAEs did not significantly impact DFS (*p* = 0.407), suggesting that early cessation of adjuvant therapy does not necessarily compromise disease control, possibly due to the well-known ICIs’ prolonged effects [28].

Our study provides further real-world evidence supporting the use of adjuvant pembrolizumab in localized/locally advanced ccRCC patients at intermediate-high or high risk of relapse, reinforcing its role as the only ICI to date with statistically significant DFS and OS benefits. Nonetheless, a subset of patients remains at higher risk of recurrence, particularly those with lymph node involvement or sarcomatoid features. This highlights an urgent need for novel biomarkers to refine patient selection and optimize therapeutic strategies. Recent attention has been directed toward kidney-induced molecule-1 (KIM-1), which has emerged as a potential biomarker for predicting the efficacy of adjuvant immunotherapy and a prognostic factor in the IMmotion010 study involving the programmed cell death-ligand 1 (PD-L1) inhibitor atezolizumab [29]. Future studies should focus on identifying predictive factors for long-term responders and evaluating the long-term impact of pembrolizumab.

To date, our study represents the largest real-world study on adjuvant treatment of renal cell carcinoma with pembrolizumab.

Recently, Mattigk A et al. presented retrospective data from a multi-institutional cohort of 52 patients, in which the recurrence rates in the M1 NED group remained high and occurred earlier compared to KEYNOTE-564 [30].

Additionally, at the 2025 ASCO Genitourinary Cancers Symposium, Uzzo R et al. presented a real-world analysis of 178 patients with non-metastatic localized RCC who underwent nephrectomy, of whom 118 received adjuvant treatment with pembrolizumab. The data were consistent with those from KEYNOTE-564 and similar to our cohort [31].

The current study is limited by its retrospective nature and the relatively short follow-up period, which may underestimate long-term recurrence rates. Additionally, potential selection biases inherent to real-world datasets should be considered. Nevertheless, the large sample size and multicentric nature of our analysis strengthen the validity of our findings.

## Conclusions

This real-world analysis supports the efficacy and safety of adjuvant pembrolizumab in resected ccRCC at an intermediate-high or high risk of recurrence, reinforcing its role as the only ICI with proven DFS and OS benefits in this setting. Despite its effectiveness, a subset of patients remains at high risk of relapse, highlighting the need for improved patient selection strategies. The identification of novel biomarkers may help refine treatment decisions. Further research is needed to optimize therapeutic approaches and assess long-term outcomes.

## Supplementary Information

Below is the link to the electronic supplementary material.

Supplementary file1 (DOCX 469 KB)

## Data Availability

No datasets were generated or analysed during the current study.
