# Supplementary material for: Real-world evidences on adjuvant Pembrolizumab for renal cell carcinoma: results from the multicenter real-world ARON-1 study
Source: Cancer Immunol Immunother. 2025 Nov 14;74(12):374. doi: 10.1007/s00262-025-04230-w (PMC12618741; doi:10.1007/s00262-025-04230-w)
Supplement: Supplementary file 1 — Supplementary file1 (DOCX 469 KB) [file 262_2025_4230_MOESM1_ESM.docx]

**Supplementary Materials**

**Figure S1.** Participating Countries.

**
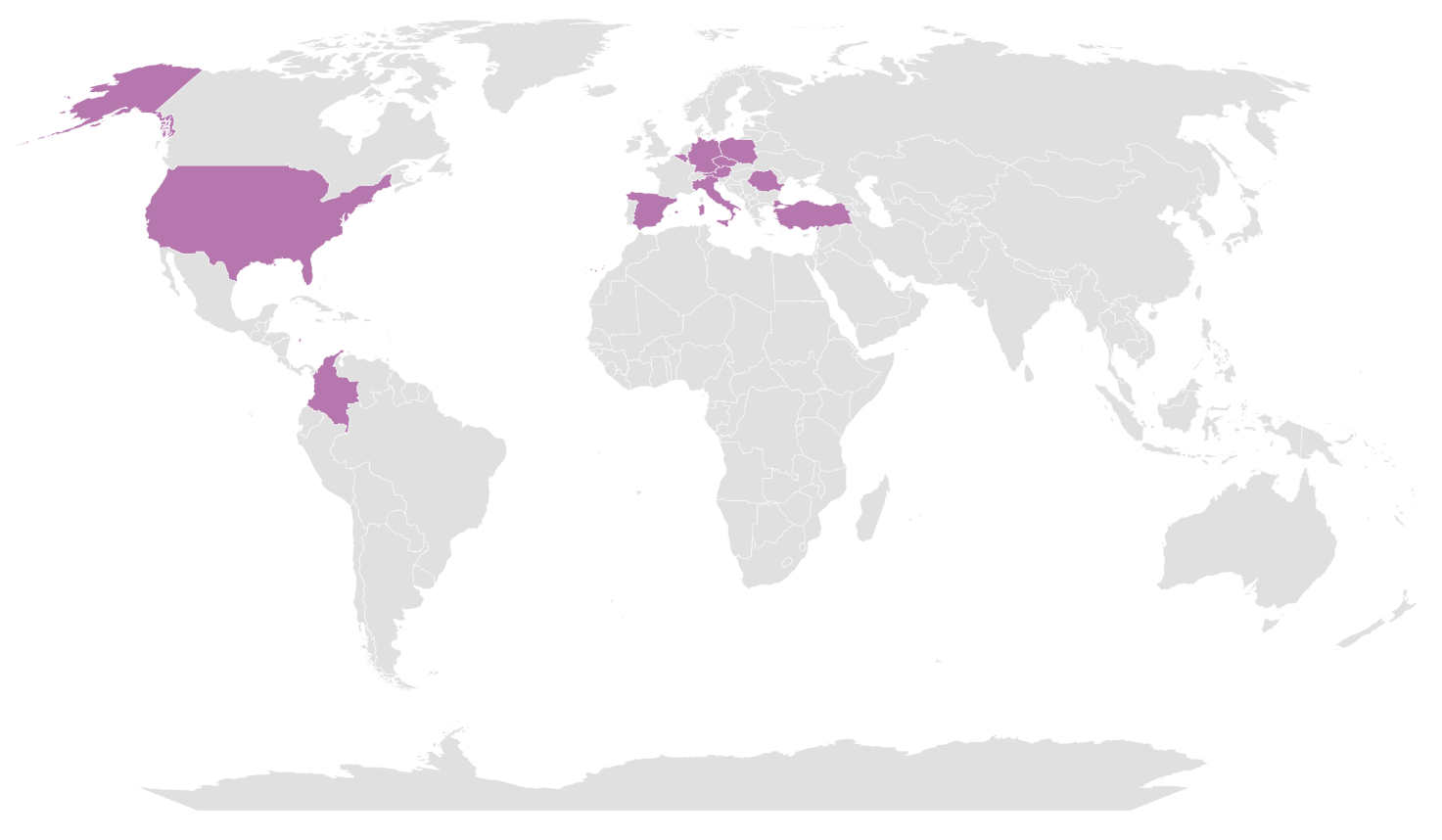
**

**Figure S2.** Selection Process from ARON-1 dataset.

**
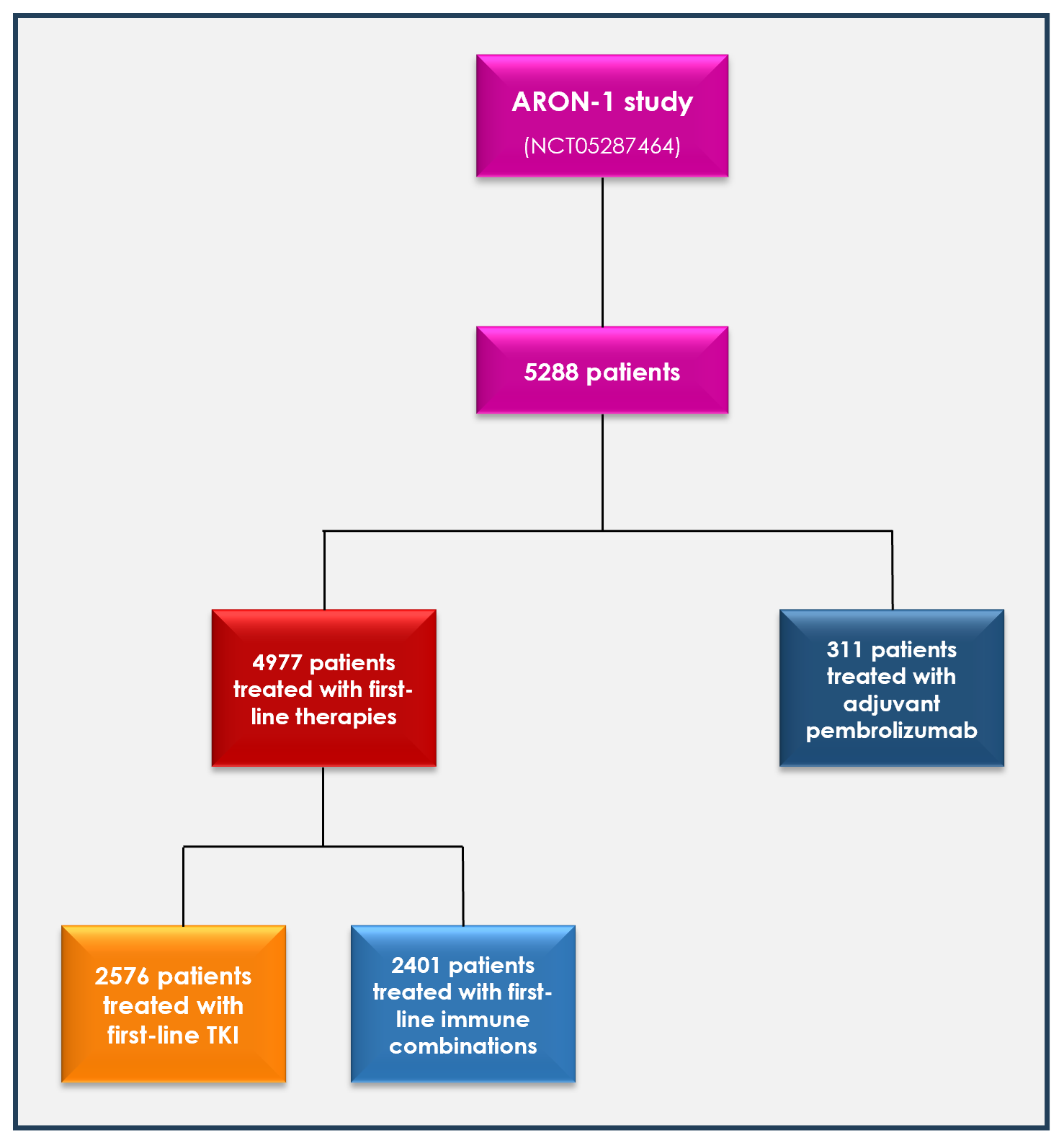
**

**Table S1**. Metastatic sites detected in recurrent patients after adjuvant pembrolizumab. Percentages refer to the overall study population.

| **Site of metastases** | **Overall**  **no. (%)** |
| --- | --- |
| **Lung** | 33 (11) |
| **Bone** | 14 (5) |
| **Distant lymph nodes** | 14 (5) |
| **Pancreas** | 10 (3) |
| **Liver** | 6 (2) |
| **Adrenal gland** | 5 (2) |
| **Brain** | 4 (1) |
| **Peritoneum** | 3 (1) |
| **Soft tissues** | 3 (1) |
| **Pleural** | 1 (<1) |
| **Thyroid** | 1 (<1) |
